# Reasons Why High Religiosity Can Co-exist with and Precipitate Discontinuation of Anti-retroviral Therapy among Different HIV Clients in Uganda: An Exploratory Study

**DOI:** 10.3390/rel3030817

**Published:** 2012

**Authors:** Christopher Tumwine, Stella Neema, Glenn Wagner

**Affiliations:** 1 Infectious Diseases Institute, Makerere University, P.O. Box 22184, Kampala, Uganda; 2 Department of Sociology and Anthropology, Makerere University, P.O. Box 7062, Kampala, Uganda; 3 RAND Corporation, 1776 Main Street, Santa Monica, CA 90407, USA

**Keywords:** religiosity, ART adherence, Uganda

## Abstract

In-depth interviews were conducted with 39 very religious people living with HIV (16 had ever and 23 had never discontinued antiretroviral therapy—ART) to assess the role of religion in these treatment decisions and in coping with HIV. Participants who had ever discontinued ART gave reasons such as: teachings and prophecies from religious leaders, and supporting Biblical scriptures all of which led them to feel that God and their faith, not ART, would help them; and testimonies by their “already healed” peers who had stopped ART. Participants who had never discontinued ART gave reasons such as continuous adherence counseling from multiple sources, improvement in physical health as a result of ART, and beliefs that God heals in different ways and that non-adherence is equal to putting God to a test. High religiosity was reported to help participants cope with HIV through engagement in personal and or community protective behaviours, “taking care of other illness”, and reducing worries. When high religiosity among people living with HIV (PHAs) becomes a barrier to ART adherence, the adherence counseling provided can draw on experiences of PHAs with high religiosity who have sustained good adherence to ART and achieved good health outcomes.

## 1. Introduction

Although religion is an important aspect in the lives of most people living with HIV (PHAs) and increases among most PHAs after being diagnosed HIV positive [1,2], many care providers (including HIV care providers) get interested in the religiosity of their clients only when it obstructs conventional treatment [3,4]. Despite this, previous research has shown that high religiosity among some PHAs can co-exist with high levels of adherence to conventional HIV treatment while among others can precipitate discontinuation of this treatment to rely only on God for healing [1]. A clear assessment of why high religiosity can (1) co-exist with non-adherence to antiretroviral therapy (ART) among some PHAs; (2) co-exist with ART adherence among other PHAs; and (3) and whether high religiosity amongst PHAs can play any positive role in their ability to cope with HIV, may help to change the perceptions of many care providers about the role of religiosity in their clients’ lives. For instance, by examining the reasons why certain PHAs are able to maintain high levels of ART adherence and religiosity at the same time, care providers will be provided important information they can use to counsel PHAs who are considering the option of discontinuing or not initiating ART as a result of religious convictions.

ART adherence is important for treatment outcomes such as viral load suppression, high CD4 count maintenance, and generally reduced morbidity and mortality among PHAs [5]. ART adherence also prevents resistance to HIV drugs thereby limiting the necessity for the more expensive second line treatment of HIV [6]. Given the numerous benefits of ART adherence to PHAs, it is important that the various barriers to high levels of adherence are clearly understood. The potential for religiosity among PHAs to influence ART adherence has been shown to exist [1,7,8].

In some studies, religiosity among PHAs has been reported to increase after HIV diagnosis [2,9]. In one study of changes in religiousness and spirituality of people with HIV/AIDS, 25% (88/347) of participants reported being more religious and 41% reported being more spiritual after being diagnosed with HIV/AIDS [9]. In a longitudinal study that examined the impact of changes in religiosity after HIV diagnosis on disease progression among 100 people, Ironson and colleagues [2] established that over 45% of the sample showed an increase in religiousness, 42% remained the same, and 13% decreased. An increase in religiosity independently predicted better CD4 cell preservation (even after controlling for several variables including antiretroviral adherence) and significantly better control of the viral load.

Studies conducted in North America such as by Guillory and colleagues [1], Kremer and colleagues [10], and Parsons and colleagues [11] report that spiritual beliefs can have both positive and negative relationships with adherence to antiretroviral therapy among different groups of people. Guillory and colleagues [1] studied religiosity and ART adherence among 45 women diagnosed with HIV and found that many of these women increased their practice of prayer and/or meditation after diagnosis; however, while some of these participants used prayer in conjunction with their conventional medical treatments, others committed themselves only to spiritual development and no longer tolerated the conventional means of treating HIV. A study of 18 peri-natally infected youth that measured religious beliefs and practices of these youths, found that participants who had excellent adherence had significantly higher religious beliefs scores and greater religious practice scores than those who had poor adherence [12]. Conversely, another study of 204 participants in the context of a controlled adherence intervention trial which partly measured religiosity in terms of engagement in religious services/practices found that high religiosity was negatively associated with ART adherence [13]. Studies conducted in Uganda have also reported mixed findings. Kisenyi and colleagues [7] in a study that measured religiosity in terms of frequency of attendance at religious services found that high religiosity was associated with high levels of adherence to anti-retroviral therapy. But in an earlier prospective observational study of 558 naïve patients starting ART in Kampala, Wanyama and colleagues [8] reported that 6 participants discontinued ART as a result of “the belief that they had been cured of HIV infection after prayers by religious leaders.”

The studies described above suggest that the mixed findings regarding the effects of religious beliefs on ART adherence rests largely in the nature of the beliefs. Conventional beliefs in God as a source of comfort, support and aid in coping with stressors appear to be associated with better adherence, while more fundamentalist beliefs in the powers of God to heal lead some patients to discontinue ART. However, it is important to note that the reported data on the negative effects of religious beliefs on adherence were based largely on anecdotal accounts of only a few respondents, rather than an established empirical relationship. Assessments of religious practices, such as frequency of religious service attendance, have consistently found positive correlations with adherence.

With the literature revealing mixed effects of high religiosity on ART adherence, we set out to examine why some PHAs with high religiosity opted to retain high levels of ART adherence, while others opted to rely only on their religious beliefs and as result discontinue ART. The aim of the analysis was to examine the reasons for these varied relationships between high religiosity and adherence to anti-retroviral therapy among people living with HIV in Uganda. This kind of analysis might pave way for designing appropriate interventions that account for both the supportive and the unsupportive roles that high religiosity can play in ART adherence.

## 2. Methods

### 2.1. Study Design and Sample

We used a grounded theory approach to investigate the reasons why high religiosity can co-exist with adherence and non-adherence to ART among different PHAs and how high religiosity helps PHAs to cope with the experience of living with HIV. Data were collected from November 2010 to January 2011 using a semi-structured interview guide. A person was selected to participate in the study if they were: HIV positive, 18 years and above, self reported high religiosity (attended 3 or more religious services in past month) and had ever started ART (however, we did not inquire from the participants about which drug regimens they were taking and for those that had discontinued treatment, which drugs they were taking before they discontinued treatment). Two categories of respondents were purposively sought for interviews; the first category was PHAs that had ever stopped ART as result of religious convictions and the second category was PHAs who had never stopped ART due to any religious convictions. Study participants were identified through the Infectious Diseases Institute, a large HIV clinic in Kampala. PHAs who reported high levels of religiosity, measured through attendance of religious services (attended 3 or more religious services in the past month) and had previously taken their medications without faith related interruptions were purposively selected and requested to participate in the study as they waited for their medical appointments. Attendance of religious services has been shown by past research such as by Edewor [14], and Guillory and colleagues [1] to be a strong predictor of the existence of other aspects of religiosity (such as saying frequent personal prayers, stronger belief in the supernatural, meditation etc) in an individual. A clinic staff member introduced the study every morning (for 14 days) to PHAs in the clinic’s waiting room (approximately 150 PHAs in attendance on average per day) and those who were interested were referred to the study interviewers for consent procedures. A list of participants who were believed to score high on religiosity but had dropped out of care/discontinued ART, was compiled in consultation with clinic counselors, and visited in their homes (first by the clinic’s home visitors during their regular home visits) to introduce the study to them. For this sub category of PHAs, only those that resided in a distance of 15 km from the Infectious Diseases Institute HIV clinic were recruited to participate in the study. The home visitors helped to identify PHAs who had opted for divine healing and as a result had dropped out of conventional care. Home visitors are counselors who visit PHAs who have stopped attending clinic to address barriers to ART adherence and continuity in care. The home visitors informed PHAs who had discontinued ART about the study; PHAs who agreed to participate in the study were visited by the study interviewers and interviewed after signing consent forms. A list of 12 PHAs was developed and all were informed about the study by the home visitors; but one of them had to be excluded from the sample because she emphasized that drug side effects rather than religious convictions compelled her to discontinue treatment. Although the other two identified PHAs confirmed to the counselors who initially visited them that religious convictions played a role in their decision to discontinue treatment, they did not like the idea of participating in the study and were thus never visited for interviews.

### 2.2. Interview Protocol

For HIV positive persons with high levels of religiosity who had ever discontinued but later resumed ART and those who were not on ART by the time of the study, we sought to understand what motivated them to discontinue ART when did so. The major research question for this group of participants was: “Why did you opt to rely only on faith for your healing?” And for those with high levels of religiosity but who continued with ART un-interrupted, we sought to understand what motivates them. The major research question for this group was: “Why didn’t you rely only on your faith for your healing?” Both categories of participants were asked: “How does your high religiosity enable you to cope with being HIV positive?” The study protocol was developed in English first, and then translated by a professional translator into Luganda (the main language in the study settings). Another professional translator, back translated the protocol from Luganda into English. All the inconsistencies between the first and the second translation were discussed with the two translators and resolved before the interviews were conducted. The interviews were conducted mostly in participants’ local language (*i.e.*, Luganda), taped, transcribed and translated into English. Few interviews were conducted in English (with participants who had higher levels of education, who could speak English fluently). The study protocol was reviewed and approved by Makerere University’s Faculty of Medicine Research and Ethics Committee and Uganda National Council of Science and Technology.

### 2.3. Data Analysis

To identify themes elicited from the interviews, text management software (*ATLAS.ti*) was used to mark contiguous blocks of transcript text that pertained to the major topical domains of interest (motivations for discontinuing ART, continuing with ART and the role of religiosity in coping with HIV). We pulled out all text associated with a particular domain and after printing the quotes on slips of paper, the team members sorted the quotes into piles based on their thematic similarities. This process involved having each member of the research team first identifying categories under each of the major themes (motivations for discontinuing ART, continuing with ART and the role of religiosity in coping with HIV). The categories developed by each member were compared. Differences between the categories were identified and the categories revised to develop more inclusive categories. After this—under each major theme, we assigned all the responses to the identified categories.

The sample characteristics of the 39 respondents are presented in Table 1: 23 had never discontinued ART, while the other 16 had ever discontinued but later resumed ART (7/16 had previously discontinued ART but resumed taking it by the time of the study, while 9/16 had discontinued ART by the time of the study). The majority of the sample was female, identified as Pentecostals, and had attended 5 or more religious services in the month before the interview.

## 3. Results

### 3.1. Reasons Why High Religiosity Can Precipitate Non-adherence to ART

The 16 study participants who had ever discontinued ART as a result of religious convictions (including those not on ART by the time of the study), gave a total of 29 reasons to explain their actions—with some participants giving multiple reasons for their decision to discontinue treatment. We grouped the 29 reasons into 4 themes—which are presented in Table 2:

#### 3.1.1. Unwavering Faith in God

Some of the participants who had discontinued ART referred to a strong faith in God, and that they can be healed without taking antiretrovirals (ARVs). Although this theme can summarize all the various themes that emerged out of the participant’s explanatory narratives, we felt this could be a stand alone theme to explain why there had been discontinuation of treatment. In relation to this theme for instance, a Pentecostal participant when asked why she was discontinuing treatment pointed out: “*I believe God will heal me; miracles used to happen and I am waiting for my miracle*.” Another Pentecostal participant stated: “*I am taking a step of faith, I want to see God’s work in my life. I am this desperate, I want to be a testimony to other people*.” Another Pentecostal participant who had previously discontinued ART, when asked what had motivated him to discontinue the treatment, pointed out: “*I had continued to pray hard, hoping that God can heal me until 2008 when I saw my life deteriorating, I was losing weight, infections were coming and taking so long to heal and that is when I went for another test which turned out HIV positive again. After that I realized I can’t rely only on faith to survive*.” Another Pentecostal participant, when asked what motivates him to rely only on faith for his healing, pointed out: “*Redeeming the spirit is the most important. My body may be sick but my spirit is not sick, because the spirit cannot get HIV. In fact I have learnt to separate the two*.” Another Pentecostal participant said: “*If you really commit yourself to prayer and pray for something from God you finally get it—that is what I am trying to do*.”

#### 3.1.2. Teachings and Prophecies from Religious Leaders

Some of the participants who had ever discontinued but later resumed ART and some of those who were not on ART by the time of study, reported that they had received prophecies that they would be healed from HIV from fellow “faithfuls” and/or church leaders. This encouraged HIV positive persons to discontinue ART and increase their religiosity through constant prayer and faith that God will heal them and wait for such a time when they would test HIV negative. Similarly, when asked what motivated them to discontinue treatment, a Pentecostal participant pointed out: “*A person with prophetic anointing who by the way was a stranger to me, told me that, ’you are going to get healed of HIV’, and so I decided to take this step of faith and believe God, it is my decision alone. Since we were strangers to each other, there is no way I could not believe this prophecy*.” Another Pentecostal participant who was asked what motivated her to discontinue treatment, pointed out: “*Three months after knowing that I had HIV, I went to the pastor, he counseled me and told me that with faith even HIV can be cured*.” Another Pentecostal participant who had discontinued treatment by the time of the study, when asked what motivated them to do so, pointed out: “*The pastor counseled me, prayed for me, comforted me and told me, you are not going to die; let us pray to God and he will heal you of HIV*.” Another Pentecostal participant who had previously discontinued treatment said: “*The pastor told me, God heals all diseases; there is nothing he cannot heal. So I spent 2 years without getting treatment waiting to be healed*.”

#### 3.1.3. Supporting Biblical Scriptures

Some of the study participants reported that a number of religious writings from the Bible encourage them to depend only on faith in God for healing rather than take what they perceive to be less effective ARVs. For instance, when asked what convinced her to stop taking ARVs, a Pentecostal participant pointed out: *“Whose reports shall we believe? It is not that I don’t respect the doctors, but they are just human beings. But we have a great Physician—who is Jesus. The bible says that by his stripes, we are healed. During his time, the lame walked, even the dead rose, and as he was going back he promised that those who decide to be his disciples, he would do much more*.” Another Pentecostal participant who was not on ART by the time of the study, when asked what motivated her to abandon treatment, pointed out: “*The bible says, if you believe without doubting, you can command the mountain to dissolve and it becomes flat. Those are the powers that Jesus left us with. By standing in those powers I believe it’s only God who knows me, he knows very well that I did not look for this problem*.” Another Pentecostal participant who had ever discontinued treatment, when asked what motivated her to stop treatment, pointed out: “*I continued praying to God with the hope that I can get cured, because, if you read the bible, it says, God can heal us*.”

#### 3.1.4. Testimonies by the “already healed” Peers Who Had Stopped ART

Some of the participants, who had discontinued ART, reported hearing and believing testimonies from persons that had already been “healed” of HIV/AIDS as result of their faith. These testimonies from fellow believers convinced some participants to discontinue treatment and only rely on spiritual growth for their own healing. In relation to this, a Pentecostal participant, when asked why she had discontinued treatment, pointed out: “*I want to be a testimony for other people to see me and know that God can heal them. By the way these kinds of testimonies where people have been healed of HIV are there; there are even doctors who are witnesses to these testimonies; it is only that they don’t want to put it at the fore-front, but the healings are happening. People are being healed, and I believe I can be one of them*.” Another Pentecostal participant when asked how being very religious has helped her, now that she is HIV positive, pointed out: “*It has helped me a lot; I usually go to my pastor he counsels me, because he also went through a similar situation. He told me, he was once sick [HIV positive] but God healed him, he even showed me scars and the rashes on his body that HIV/AIDS left him with*.” Another Pentecostal participant when asked what had motivated her to discontinue ART, pointed out: “*My Pastor was sick (HIV positive) too, but God helped him and he got healed; so he told me to pray until that time when I will test and find that I am negative*.” Another Pentecostal participant who had ever discontinued treatment, when asked what motivated her to discontinue ART pointed out: “*My cousin convinced me to go to her church and pray. On getting there, I saw people giving their life testimonies and amongst those giving testimonies were people who were saying they were healed of HIV. So I decided to leave the drugs because I thought I had obtained an effective cure*.”

### 3.2. Reasons Why High Religiosity Can Co-exist with High ART Adherence

The 23 study participants who had never discontinued ART as a result of their religious convictions gave a total of 37 reasons to explain their actions, with some participants giving multiple reasons for their decision not to discontinue ART and rely only on their faith for healing. We grouped the 37 reasons into 6 themes which are presented in Table 3:

#### 3.2.1. Perceived Inefficacy of only Prayers to Heal HIV

Some study participants who reported high levels of religiosity and high ART adherence levels felt that because they have never seen anyone who has been healed after receiving prayers, there is no need to only rely on prayers to be healed of HIV. A Pentecostal participant who reported she had a stomach ache for years and the pastor prayed for her and she got healed, when asked whether there are diseases that pastors can successfully heal through prayers while for others they can’t, pointed out: “*I have seen mad people being healed by Pastors’ prayers, but I have never seen any HIV+ person being healed, that is why I could not ask him to pray for me to heal from HIV*.” Another Pentecostal participant who reported disclosing her status to her religious leader when asked what the leader advised her about ARVs after learning about her status, pointed out: “*Although he advised me to commit myself only to prayers for a cure, I know there is no pastor who can heal me of HIV. I know that the medicine I have or the medicine that will come in future can only help me. I only trust doctors. Pastors have never healed anyone of HIV*.”

#### 3.2.2. Improvement in Physical Health as a Result of ART

Some participants emphasized that improvement in their own physical health after continuously taking ARVs for sometime had enabled them to grow stronger in faith. For instance, a Pentecostal participant who had decided not to hear anything about God after being diagnosed with HIV, but later got stronger in faith pointed out: “*In 2004 I was tested and my CD4 count was very low (at 9), then I was started on ARVs and within 4 months my CD4 count had risen to more than 300; then, I realized that God still needed me to do many other good things here on earth. I decided to double my efforts in praising God, taking my medication and doing all the good things that God wants me to do*.” A Protestant participant when asked why she has not relied only on faith for her healing, pointed out: “*Although I was not physically down the very first day I came to the clinic, I was started on drugs [ARVs] because my CD4 count was low; so, for this improvement, I doubled not only my faith in him but also my adherence to drugs*.” Another Pentecostal participant when asked why he has not relied only on God for his healing, pointed out: “R*ecently they checked my viral load, and they said they could not detect the virus; this, helped me to know that there is a super natural some thing; I thank God for this*.”

#### 3.2.3. Continuous Adherence Counseling from Multiple Sources

A theme that came up frequently was that adherence counseling from multiple sources has enabled many participants to continue on treatment while remaining very religious at the same time. Adherence counseling was reported to come from numerous sources such as clinic counselors, family members, friends and even religious leaders. In relation to this, in response to a question on why they have been able to maintain high levels of adherence to ART and religiosity at the same time, a Pentecostal participant pointed out: *“The counselors continue to guide us. They even came to my home and told me that if I leave the medicine [ARVs] and die, it will be my fault*.” Another Pentecostal participant when asked whether the religious leader has ever advised them to discard ARVs in favour of spiritual healing, pointed out: “*No, in fact the counseling he was giving us was that now, that you know your status, you should keep your selves pure. I remember he even asked us where we were getting our medications from*.” Another Pentecostal participant pointed out: “*When I got to my Pastor he told me, HIV used to be a death sentence; so who ever contracted it, was supposed to die, but it is no longer so, since the drugs have been found, these can help you live as long as you want*.” A Catholic participant who carried ARVs and camped at the local church to pray for her condition, when asked what the religious leader advised her about taking ARVs, pointed out: “*They told us to come with our medicine (ARVs) as they pray for us. They told us to first go to the hospital and come back to Jesus*.” Another Pentecostal participant who disclosed to his religious leader immediately after receiving his test results, when asked how the leader responded, pointed out: “*He consoled me and comforted me, in fact he is the one who brought me here to this clinic to start on treatment*.” Apart from some religious leaders, the other promoters of ART adherence were reported neither to vigorously encourage nor discourage spiritual growth alongside the use of conventional HIV medications.

#### 3.2.4. God Gave Us Wisdom and Some Level of Autonomy

A theme that also came through the interviews was that although human beings are dependant on God, they were given wisdom and autonomy to help chart their destiny. They are given knowledge and wisdom to be able to act independently, and change their well-being. Developments in the field of anti-retroviral therapy are seen as a result of the use of the wisdom that God provides to human beings. When seen in this light, the use of ART is presented as reinforcing the purpose of God. In relation to this, a Protestant participant when asked whether the use of ART does not mean that they don’t trust God, pointed out: “*God does not want us to sin but for us we go ahead and sin, and God heals but he passes through doctors; …So if you don’t take medicine it’s like you are killing your-self*”. Another Protestant participant who was thankful to God for improvements in her physical health, when asked whether the changes she was seeing in her life were being brought by the medicine or God, pointed out: “*It’s God who gives the Doctors knowledge to bring this medicine. Now, I have hope that one day even a total cure will come; with God’s guidance, one day scientists will wake up and tell us that we have got the medicine*”. A Catholic participant who after being put on ARVs went and camped at the church for two months to pray, when asked why she had to go with the ARVs to church pointed out: “*Among the Catholics we say, if an enemy attacks you don’t sit down and watch and think that God will help you, you first need to defend yourself and thereafter seek God’s guidance*.” A Pentecostal participant when asked why he takes medication regularly when they could believe that God will heal them, pointed out: “*I believe that God is a healer, but I also believe that God is the one who gives men wisdom, so if men have come up with the idea of ARVs and they are working, I have to use them*”. Another Pentecostal participant when asked why they don’t rely only on the belief that God will heal them, pointed out: “*I believe that he can heal me but even Pastors go to hospital when they fall sick; so why not me—the sheep, who is supposed to follow them?*”

#### 3.2.5. God Heals in Different Ways

From the participants’ narratives on why they have been able to sustain high levels of religiosity and ART adherence side by side, a theme emerged on God’s ability to heal people in different ways. Participants talked of Jesus healing the sick through just touching on them, while for others he used words, and others –he told them to go and wash themselves; and they were all healed. These various ways through which healing was provided, create feelings among some participants that using anti-retroviral therapy is one of those ways through which God can heal them. For instance, when one Catholic participant was asked whether it is not a contradiction to pray for healing from HIV as she takes the ARVs, replied: “*What I think is that, prayer and medication (ARVs) go together. You pray and ask God to let the medicine work for you. Both go hand in hand*.” Also when a Protestant participant was asked whether it is not inadequate trust in God that convinces her to continue taking medication, pointed out: “*In our church we have a belief that: medication + prayers = life*.” When the same participant was further asked whether if one says only prayers equal to life, what would happen, she pointed out: “*Things may not work; we believe that God helped the scientists, to discover the drugs that we currently have*.”

#### 3.2.6. Non-adherence to ART is Equal to Putting God to a Test

Out of the data emerged a theme which emphasized that non-adherence or discontinuation of ART to rely only on faith in God for a divine cure is equal to putting God to a test. All the participants who echoed this theme did not find it conceivable that God should be put on a test at any time. For instance, when asked if his faith would not be regarded as low if he continues to take ARVs, a Pentecostal participant remarked: “*The bible says don’t put your lord God on a test; he has never stopped any one from going to hospital. If you stop taking medication and your health fails, what now happens to your faith in God after that?*” Another Pentecostal participant when asked whether praying to be healed of HIV while taking ARVs isn’t an act of little faith, pointed out: “*It is cultic churches which teach that way; for example if you teach that Jesus fasted for 40 days, so, you should do the same, that is not possible! You cannot arm twist God, by saying that if he does not do this, you will also not do that. God works in his time; he can even heal those who have not prayed, like the beggar Jesus healed on the gates of Jerusalem*.”

### 3.3. High Religiosity as a Mechanism for Coping with HIV

Nearly all participants reported their religiosity increased after being diagnosed HIV positive. We asked both categories of participants how high religiosity was helping them to cope with HIV, and the responses from the two groups of participants fell into these common themes.

#### 3.3.1. Engagement in Personal and Community Protective Behaviours

Both categories of participants frequently talked about the increase in their religiosity helping to reduce and sometimes to eliminate self-destructive behaviours such as engagement in alcohol use, smoking, and multiple sex partners. Both categories of participants reported noticeable changes in their lifestyles, changes that involve abandoning behaviours that are considered to be for the non believers. When a Pentecostal participant was asked how the increase in his religiosity was helping him cope with HIV, he pointed out: “*It has helped me a lot because before I got saved I was a drunkard, a womanizer and used to smoke a lot; if I had stayed with that kind of life style, I would have died long time ago. …Because of that lifestyle, money could never settle in my pockets; but now, I can be able to pay [school] fees for my children*.” A Protestant participant pointed out; “*Being religious enabled me to stop drinking alcohol; given my HIV status, I don’t think if I had continued with my drinking rate, I would still be in this world*.”

A few participants reported that their religiosity compelled them to engage in community HIV protective behaviours such as limiting their own number of sexual partners in order not to spread HIV and encouraging their social network members to either abstain from sex or be faithful to their partners. In relation to this, when asked whether being very religious has helped her in dealing with HIV, a Muslim participant pointed out: “*It has helped me a lot because I have learnt that there is need to help others not contract HIV; I have to disclose my status to help others, for example, I am still looking good and men still approach me for sex. So, because I am strong in my faith, I can’t infect them. It is like committing murder! I disclose my status to whoever relentlessly tries to woo me*.”

#### 3.3.2. “Can Take Care of Other Illnesses but not HIV”

Both categories of participants reported that their high religiosity helped them in taking care of their other illnesses. Many participants that had never discontinued ART, repeatedly mentioned reliance on a higher power to heal them of their other illnesses. One such Pentecostal participant that had never ever discontinued ART, when asked what her high religiosity was helping her, pointed out: “*I have got a lot in being born again. I was having a serious disease in my stomach but the pastor prayed for me and it went away*”. When further asked why she did not ask the same pastor to pray for her to heal from HIV, she pointed out: “*There are diseases that pastors can handle and HIV is not one of them. So, if the pastor tells me to stop taking ARVs, I cannot stop*.” But, for some participants that had previously discontinued ART, the conviction that reliance on faith only would eventually heal them of HIV seemed to also apply to other diseases they sometimes have to deal with. A Pentecostal participant who had discontinued ART when asked how being religious was helping him, pointed out: “*God is a healer, I am relying on him to take care not only of my current condition but also the many other health problems that confront me at times*.”

#### 3.3.3. Better Mental Health/Reducing Worries

Both categories of respondents reported that the increase in religiosity reduced their worries about the possibility of dying, and reduced their depression. Participants, who were not on ART by the time of the interviews seemed to emphasize being less worried about death than their other HIV-positive peers. When asked how her faith was helping her cope with being HIV positive, one Pentecostal participant said: “*I no longer worry about the possibility of dying, because the road to where I will go after here is now straight and very clear to me*.” Another Pentecostal participant who had previously discontinued ART, stated: “*When I feel very low, I talk to God and I get lifted up*”. Another Pentecostal participant when asked how his high religiosity was helping him to cope with HIV, pointed out: “*Before getting saved, I used get into situations that would make me feel bad—I would feel bad when I would be very broke and without any money, but now, I keep on trusting God that he will provide. For example, when I don’t get the food I want to eat, I say God will give it to me, instead of worrying so much. Now I have settled thoughts*.”

### 3.4. Discussion

Our study findings suggest that it is possible for PHAs to be both highly religious and be very adherent to ART. Our findings also further suggest that it is possible for high religiosity to lead to discontinuation of ART among other PHAs. Various reasons are cited by each of the two groups to explain their decisions. On one hand PHAs who had high religiosity and ART adherence, gave reasons such as perceived inefficacy of prayers to cure HIV, continuous adherence counseling from multiple sources, beliefs such as discontinuing treatment is putting God on test, and God heals in different ways. On the other hand, PHAs with high religiosity, but who had discontinued ART, gave reasons including supporting biblical scriptures, teachings and prophecies from religious leaders, and testimonies by “already healed peers” who had stopped ART to explain their decisions.

Although our sample does not allow us to draw any conclusive statements on the influence of differences in religious affiliation on continuity in conventional HIV care, our predominantly Pentecostal sample suggests that the evangelical/‘born again’ religious groups may be more prone to being less supportive of conventional medicine, and this should be explored further in future research with a wider representation of various religious traditions. All the participants (16/39), that reported to have ever, or were not on ART during the time of the study were Pentecostals. But, we also note that, not all the Pentecostals that were interviewed in the study reported to have ever discontinued ART due to religious convictions or any other reasons.

The study findings suggest that it is possible for high religiosity among PHAs to obstruct and to co-exist with conventional HIV treatment within a single individual at different time periods. A good number of PHAs (7/39) that were recruited from the clinic reported that they had ever discontinued conventional HIV treatment due to convictions that they would be healed of HIV by use of prayers, but only to realize later this could not be possible. This implies that with a clear understanding of why some PHAs are able to return for ART, conventional health care providers can rely on this information to help future PHAs that choose to rely only on prayers for their healing rather than enrolling into HIV antiretroviral therapy programs. This finding that some PHAs can return for ART after opting for prayers only, is also consistent with findings from Wanyama and colleagues [8] where it is reported that the majority of PHAs that had discontinued ART due to a belief in divine healing had resumed it after sustained counseling by care providers.

The current findings suggest that high religiosity among PHAs can help them in coping with the otherwise stressful condition of living with HIV. Study participants reported that high religiosity enabled them to reduce their worries about the possibility of dying. Other participants reported that high religiosity enabled them to cope with HIV through taking care of other illnesses that at times confront them, and also by encouraging study participants to engage in personal and community protective behaviours. The study finding that high religiosity reduced participants’ worry about the possibility of dying is consistent with findings from other writers such as Koenig [15], Koenig and colleagues [16], Cummings and Pargament, [17], Dalmida [18], de Jager Meezenbroek and colleagues [19], Kagimu and colleagues [20] who report that religion can offer comfort in situations of stress.

There are a number of limitations to the study. The sample size was small, comprised of only those who were interested in the study, and because of this, it may be difficult to generalize the findings to all HIV positive persons receiving care in Uganda. All respondents were recruited from an urban setting in Kampala and this makes it hard to generalize the findings from this sample to populations in rural Uganda. Almost all respondents were Pentecostal Christians, and this sample may not be representative of other religious denominations that were not adequately represented in the study. Also, religiosity was measured using attendance of religious services; there might be some very religious persons who do not regularly attend religious services and were thus left out of the study.

Future research should focus on those PHAs who drop out of conventional HIV care as result of religious convictions and finally return to restart ART. A clear understanding of the major influences and the decision processes they undergo to finally return to restart ART may help to design information packages to help those that have dropped out or about to drop out of care due to religious convictions. There is also need for further longitudinal studies that can examine whether systematic passing on of religious information that supports continuity in conventional HIV care, can help to reduce drop out rates from conventional HIV treatment programs due to religious convictions or not. Since this exploratory study is among the first to examine why some PHAs with high religiosity discontinue ART while others do not, we suspect there could be more reasons for this in addition to the themes reported in this paper. More research on this particular subject is needed to exhaustively uncover the explanations for these two scenarios.

## 4. Conclusions

In conclusion, our findings suggest that high religiosity among PHAs can help them cope better with the experience of living with HIV, and that it is possible for high religiosity to be either supportive of high ART adherence levels or a barrier to ART adherence among some people living with HIV. When religiosity among PHAs becomes a barrier to ART adherence, conventional care providers should recognize that high religiosity can co-exist with high ART adherence among many other PHAs, and draw on experiences of their own patients or the experience of the participants quoted here to counsel patients considering opting for divine healing over conventional treatment. Counseling of these patients could employ persuasive religious reasons for continuing with ART, as illustrated by quotes from our study participants: “*God heals in different ways*”, “*God gave human beings some level of wisdom and autonomy, that we can utilize in times likes these*”, “*Discontinuation of ART can be equal to putting God on a test*”. In cases, where the medical care provider does not feel well qualified to put together a convincing religious explanation that can possibly enable a PHA (with reservations about ART) to continue with ART, a referral system to religious leaders who appreciate the role of adherence to ART could be useful. Such religious leaders need to be equipped with relevant basic information about HIV treatment as recommended by care providers so that they do not mislead PHAs.

## Figures and Tables

**Table 1:** Study Participants’ characteristics (n = 39).

Participant characteristic	Number (%)
**Gender**	
Male	6 (15.4)
Female	33 (84.6)
**Age**	
20–29	7 (18)
30–39	10 (26)
40–49	15 (38)
50–59	5 (13)
60–69	2 (5)
**Education**	
Primary school or less	7 (19)
Secondary school (S.1–S.4)	15 (38)
Secondary School (S.5–S.6)	6 (15)
Diploma up to University	11 (28)
**ART status**	
Had ever discontinued but later resumed ART	7 (18)
Not on ART by time of the study	9 (23)
Never discontinued ART	23 (59)
**Religious affiliation**	
Born Again/Pentecostals	33 (84.6)
Protestant	2 (5.1)
Catholic	3 (7.7)
Moslem	1 (2.6)
**Attendance of religious services in the past month**	
Attend 3–4 services	9 (23)
Attend 5 or more services	30 (77)
**Employment**	
Formal (salaried) employment	4 (10.3)
Self employed (in informal sector)	20 (51.2)
Unemployed	15 (38.5)

**Table 2:** Reasons why high religiosity can precipitate non-adherence to antiretrovirals (ARVs) (n = 16).

Reason	Number (%)
Unwavering faith in God	14 (48)
Supporting Biblical Scriptures	5 (17)
Testimonies by the “already healed” peers who had stopped ART	6 (21)
Teachings and Prophecies from religious leaders	4 (14)
***Total***	***29 (100)***

**Table 3:** Reasons why high religiosity can co-exist with adherence to ART (n = 23).

Reason	Number (%)
Perceived inefficacy of only prayers to heal HIV	7 (19)
Improvement in physical health as a result of ART	6 (16)
Continuous adherence counseling from multiple sources	4 (11)
***Beliefs such as:***	
God gave us wisdom and some level of autonomy	7 (19)
God heals in different ways	10 (27)
Non-adherence to ART is equal to putting God to a test	3 (8)
***Total***	***37 (100)***
